# The loop-less ^tm^Cdc34 E2 mutant defective in polyubiquitination *in vitro *and *in vivo *supports yeast growth in a manner dependent on Ubp14 and Cka2

**DOI:** 10.1186/1747-1028-6-7

**Published:** 2011-03-31

**Authors:** Agnieszka Lass, Ross Cocklin, Kenneth M Scaglione, Michael Skowyra, Sergey Korolev, Mark Goebl, Dorota Skowyra

**Affiliations:** 1 Edward A. Doisy Department of Biochemistry and Molecular Biology, Saint Louis University School of Medicine, St. Louis, MO 63104, USA; 2 Department of Biochemistry and Molecular Biology, Indiana University School of Medicine, Indianapolis, IN 46202, USA; 3 Dept. of Neurology, University of Michigan Medical School, Ann Arbor, MI, USA; 4 Dept. of Microbiology, Washington University School of Medicine, St. Louis, MO, USA

## Abstract

**Background:**

The *S73/S97/loop *motif is a hallmark of the Cdc34 family of E2 ubiquitin-conjugating enzymes that together with the SCF E3 ubiquitin ligases promote degradation of proteins involved in cell cycle and growth regulation. The inability of the loop-less ^Δ12^Cdc34 mutant to support growth was linked to its inability to catalyze polyubiquitination. However, the loop-less triple mutant (tm) Cdc34, which not only lacks the loop but also contains the S73K and S97D substitutions typical of the *K73/D97/no loop *motif present in other E2s, supports growth. Whether ^tm^Cdc34 supports growth despite defective polyubiquitination, or the S73K and S97D substitutions, directly or indirectly, correct the defect caused by the loop absence, are unknown.

**Results:**

^tm^Cdc34 supports yeast viability with normal cell size and cell cycle profile despite producing fewer polyubiquitin conjugates *in vivo *and *in vitro*. The *in vitro *defect in Sic1 substrate polyubiquitination is similar to the defect observed in reactions with ^Δ12^Cdc34 that cannot support growth. The synthesis of free polyubiquitin by ^tm^Cdc34 is activated only modestly and in a manner dependent on substrate recruitment to SCF^Cdc4^. Phosphorylation of C-terminal serines in ^tm^Cdc34 by Cka2 kinase prevents the synthesis of free polyubiquitin chains, likely by promoting their attachment to substrate. Nevertheless, *^tm^CDC34 *yeast are sensitive to loss of the Ubp14 C-terminal ubiquitin hydrolase and DUBs other than Ubp14 inefficiently disassemble polyubiquitin chains produced in *^tm^CDC34 *yeast extracts, suggesting that the free chains, either synthesized *de novo *or recycled from substrates, have an altered structure.

**Conclusions:**

The catalytic motif replacement compromises polyubiquitination activity of Cdc34 and alters its regulation *in vitro *and *in vivo*, but either motif can support Cdc34 function in yeast viability. Robust polyubiquitination mediated by the *S73/S97/loop *motif is thus not necessary for Cdc34 role in yeast viability, at least under typical laboratory conditions.

## Introduction

The covalent attachment of ubiquitin to other proteins often serves as the signal for their degradation by the 26 S proteasome [1]. Protein ubiquitination depends on a cascade of ubiquitin transfer reactions that begins with the formation of a high-energy thiolester between the C-terminus of ubiquitin and the catalytic site cysteine of the E1 ubiquitin-activating enzyme. The activated ubiquitin is transesterified to the active site cysteine of one of many E2 ubiquitin-conjugating enzymes and then conjugated to specific substrates in a manner dependent upon specific E3 ubiquitin ligases. While the term "ubiquitin ligase" implies that all E3s are enzymes, only the HECT-type E3s contain a catalytic site cysteine that directly participates in the ubiquitin transfer. The RING-type E3s promote ubiquitination of specific substrates by the catalytic site of an E2. The significance of this difference is unclear, as both types of ubiquitin transfer cascades lead to formation of an isopeptide bond between the C-terminus of ubiquitin and a lysine residue on the substrate. Protein substrates can be modified with one or multiple ubiquitins and a chain of polyubiquitin can be synthesized when a lysine of ubiquitin serves as the isopeptide bond acceptor. A chain involving lysine 48 (K48) of ubiquitin and composed of at least 4 ubiquitins serves as the primary signal for substrate proteolysis [2,3].

The RING-type SCF E3s are the largest and arguably most extensively studied family of ubiquitin ligases, with members present in all eukaryotes [4]. The discovery of the SCF E3s was initiated by the observation that the *S. cerevisiae CDC34 *gene encodes an E2 [5] that together with the Cdc53, Skp1 and Cdc4 cell cycle regulatory proteins promote degradation of the Sic1 S-phase cyclin-dependent kinase inhibitor, thereby permitting entry into S phase [6,7]. A subsequent biochemical reconstruction [8-11] showed that Skp1, Cdc53, Rbx1 and Cdc4, an F-box protein, form the SCF^Cdc4 ^E3 complex. The large number of F-box proteins with different C-terminal protein-protein interaction motifs enables the family of SCF E3s to recruit many substrates for ubiquitination by Cdc34, usually in response to substrate phosphorylation.

Of the eleven E2 ubiquitin-conjugating enzymes in *S. cerevisiae*, only Cdc34 functions with SCF E3s *in vivo*. This specificity is attributed to the unique C-terminus of Cdc34 that includes a domain necessary for binding to SCF [12-14], because Cdc34 mutants that lack this C-terminal domain cannot support yeast viability and a fusion protein containing this domain and the catalytic core of Rad6 can replace Cdc34 function *in vivo*. Based on this evidence it has been proposed that the catalytic cores of the E2s are interchangeable [15,16].

**Figure 1:**
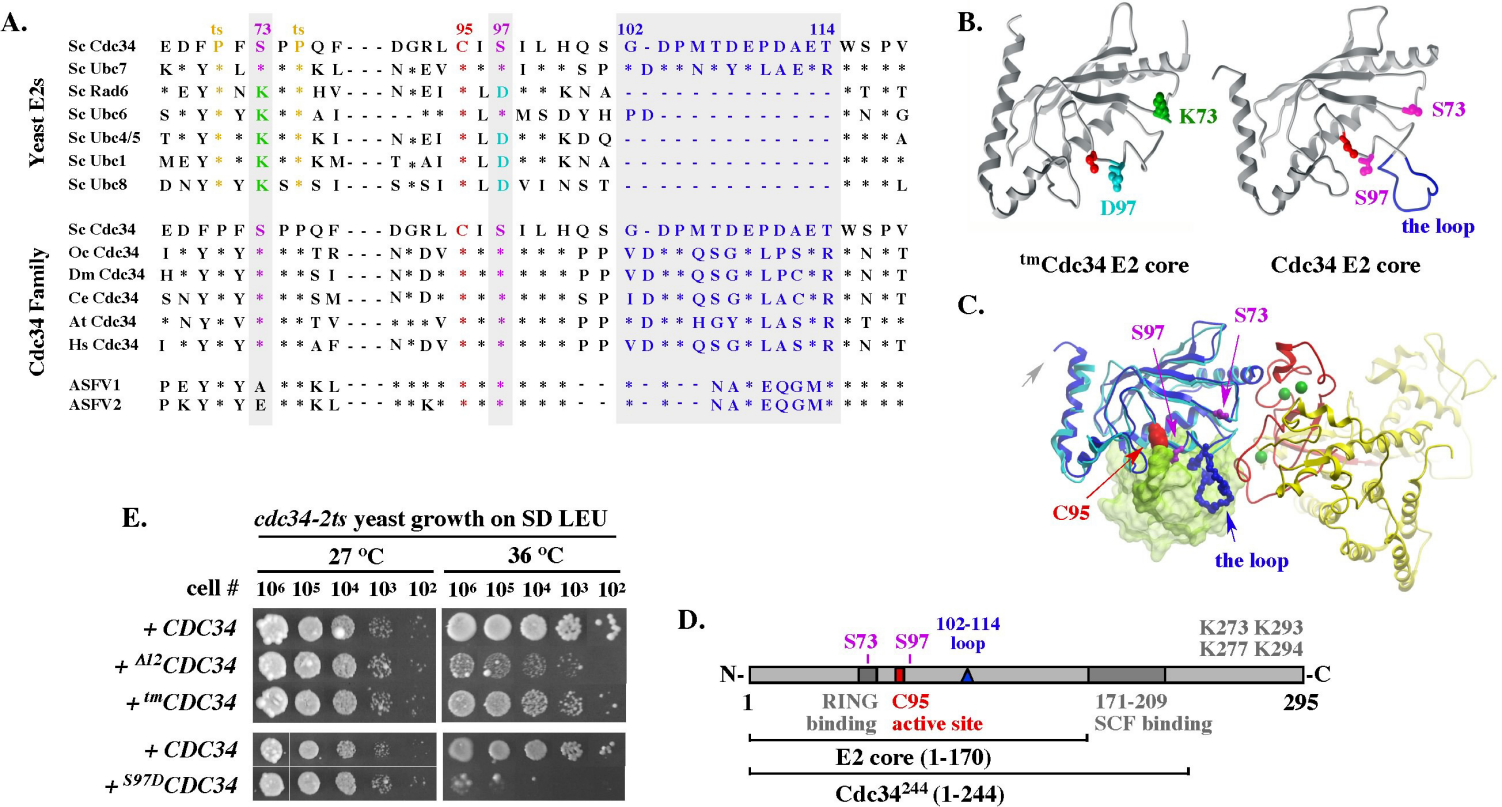
**Disruption, but not replacement, of the *S73/S97/loop *motif conserved among the Cdc34/Ubc7 E2 family is lethal for yeast**. (A). Partial sequence alignment. Asterisks represent residues identical to Cdc34 and dashes represent gaps. Sc - *S. cerevisiae*; Oc - *O*. *cuniculus*; Dm - *D. melanogaster*; Ce - *C. elegans*; At - *A. thaliana*; Hs - *H. sapiens*; ASFV1 - African swine fever virus (GI:9628248); ASFV2 -African swine fever virus (GI:450743). (B). Structural models of the E2 core (a.a. 1-170) of Cdc34 and ^tm^Cdc34. Residues corresponding to K73 and D97 in ^tm^Cdc34, and S73 and S97 in Cdc34, are color coded as in A and shown in the context of structures of a scRad6 and scUbc7 fragment (Methods); navy blue: the acidic loop formed by scUbc7 residues corresponding to amino acids 103-114 in Cdc34; red: the residue corresponding to the catalytic site C95 of Cdc34 and ^tm^Cdc34. (C). Model of ubiquitin-charged Cdc34 (a.a. 1-170) bound to the RING domain of Rbx1. See Methods. (D). Scheme of Cdc34 domains of interest. (E). Rescue of *cdc34-2ts *yeast growth with Cdc34 E2 core mutant constructs. Cultures of *cdc34-2ts *strain carrying the indicated constructs under the *GAL10 *promoter on a 2μ YEp51 plasmid were grown overnight at 27°C in SD-Leu, adjusted to a density of 1 × 10^8 ^cells/ml, serially diluted, spotted onto SD-Leu plates and incubated at permissive (27°C) or non-permissive (37°C) temperature for 4 days. Note that dextrose allows only low expression of the *GAL10 *controlled constructs.

On the other hand, three elements, S73, S97 and an acidic loop, distinguish the E2 cores of Cdc34 and Ubc7 from other ubiquitin conjugating enzymes (Figure 1A) [17]. The significance of these elements is best illustrated by the lethality associated with the substitution of residue S97 or deletion of the loop alone (Δ12) [17]. The loop-less ^Δ12^Cdc34 monoubiquitinates the SCF^Cdc4^-dependent substrate Sic1 with a rate comparable to the rate of wild type Cdc34 (0.2 pmol/s) but synthesizes K48-type di-ubiquitin at a 10-fold slower rate (0.08 pmol/s vs. 0.8 pmol/s). Based on these findings it has been proposed that the loop plays a key role in the synthesis of K48-type polyubiquitin [18,19]. Similarly, the S97D replacement prevents self-association of Cdc34 molecules [20], a step implicated in the activation of polyubiquitination [21]. An E2 protein lacking both of these elements would be predicted to be even more defective and fail to support yeast growth. Contrary to this expectation, the S97D substitution and the loop deletion act as intragenic suppressors, and the suppression is best when combined with the S73K substitution, which by itself has no phenotype. The resulting triple mutant (tm) Cdc34 (S73K, S97D, Δ12) has the *K73/D97/Δloop *motif typical of other E2s, no longer displays the self-associating defect typical of the S97D substitution alone and supports the growth of *cdc34-2ts *mutant yeast at a non-permissive temperature. A recent genetic study shows that the *^tm^CDC34 *allele supports yeast growth even when expressed from the natural chromosomal location of *CDC34 *[22]. However, this gene replacement causes numerous yet not fully understood changes in gene expression and evokes sensitivity to loss of several genes, including genes previously linked to function of the Cdc34/SCF pathway. Among these genes is *CKA2*, which encodes the catalytic subunit of casein kinase 2 that phosphorylates Cdc34 [23,24]; *RPN10*, which encodes the classic ubiquitin-binding receptor of the proteasome; and *RAD23*, which encodes the UBA-UBL type of a shuttle protein that genetically and biochemically interacts with *RPN10 *[25,26]. One of the key unanswered questions is whether ^tm^Cdc34 supports cell growth despite compromised polyubiquitination, or the S73K and S97D substitutions, directly or indirectly, correct the defect in polyubiquitination associated with the loop absence.

We show that replacement of the catalytic motif compromises polyubiquitination activity of Cdc34 and alters its regulation *in vitro *and *in vivo*. Nevertheless, either motif can support Cdc34 function in cell growth and division, with normal cell size and cell cycle profile. These findings suggest that robust polyubiquitination catalyzed in a manner dependent on the *S73/S97/loop *motif is not necessary for Cdc34 function *in vivo*, at least under typical laboratory conditions. The *S73/S97/loop *motif conservation could be associated with a yet unappreciated role of Cdc34, possibly under conditions of stress and/or compromised growth.

## Results

### Isogenic *CDC34 *and *^tm^CDC34 *yeast strains have similar growth properties, cell size and cell cycle profiles under typical laboratory conditions

Due to an essential role of Cdc34 in cell division [5], mutations that compromise Cdc34 function *in vivo *lead to cell cycle arrest. Monitoring growth and cell cycle phenotype is thus a common approach to assess the role of Cdc34 residues and/or motifs.

Previous analysis of growth phenotypes suggested that the triple mutant ^tm^Cdc34 in which the *S73/S97/loop *motif conserved among the Cdc34-like E2s is replaced with the *K73/D97/no loop *motif typical for other E2s (Figure 1A-D) supports yeast growth. This conclusion was based on complementation of the *cdc34-2 *temperature sensitive (ts) and *cdc34Δ *mutant yeast by over-expressed chimeric *^ch^CDC34/RAD6 *[16] or triple mutant *^tm^CDC34 *[17]; an approach that could mask a functional defect due to overproduction of the complementing protein. However, we obtain similar results when *cdc34-2^ts ^*yeast express the *^tm^CDC34 *allele from the *GAL1 *promoter under non-inducing conditions thereby ensuring only low level expression (Figure 1E). In contrast, growth is not supported by *^S97D^CDC34 *that carries only the S97D replacement, or by *^Δ12^CDC34 *that lacks the loop alone. Disruption of the *S73/S97/loop *motif in Cdc34, but not its replacement with the alternative E2 core motif is thus lethal for yeast.

**Figure 2:**
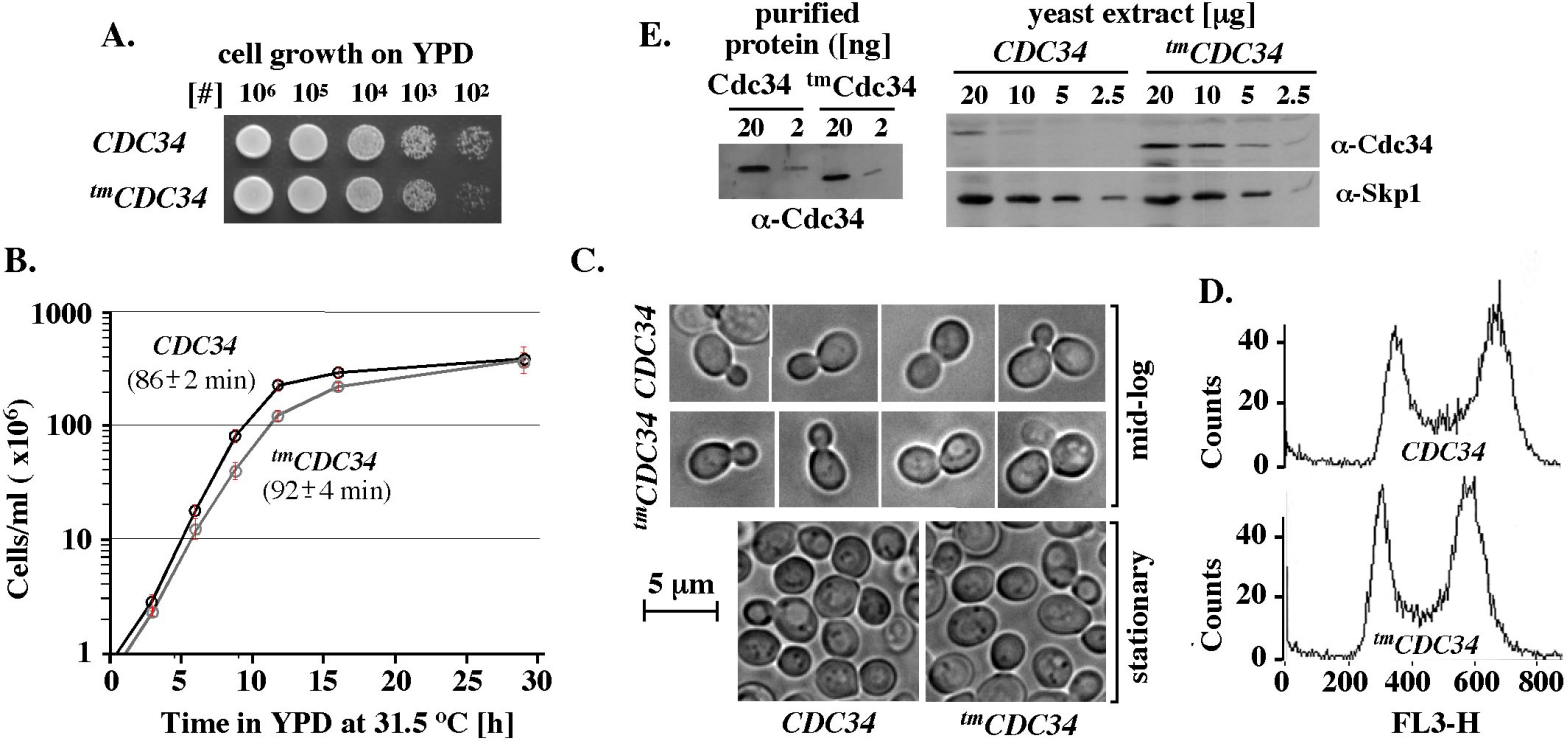
**Isogenic *CDC34 *(BL2) and *^tm^CDC34 *(RC85) yeast strains have similar growth properties, cell size and cell cycle profiles under typical laboratory conditions**. (A). Growth on plates. Overnight cultures were adjusted to a density of 1 × 10^8 ^cells/ml, serially diluted, spotted onto YPD and grown at 30°C. (B). Growth in liquid culture. Each strain was inoculated in three biological replicates into pre-warmed 50 ml of YPD at a starting density of 5 × 10^5 ^cells/ml and grown with vigorous shaking at 31.5°C. The doubling time for *CDC34 *(BL2) is 86 +/- 2 min and *^tm^CDC34 *(RC85) is 92 +/- 4 minutes during the exponential phase of growth (0-11.75 hrs). (C). Light microscopic images of cells. Yeast cultures were grown as in B, with images collected at 1.5 × 10^7 ^cells/ml (mid-log phase) and 2 × 10^8 ^cells/ml (stationary phase). (D). Cell cycle distributions. Yeast cells were analyzed by flow cytometry for their DNA content using propidium iodide staining (Methods). (E). Quantitative western blot analysis of the steady-state levels of Cdc34 and ^tm^Cdc34 proteins. Yeast extracts were prepared as described in Methods. The indicated amounts of total proteins were separated by SDS-PAGE and analyzed by α-Cdc34 WB. Control α-Skp1 WB was performed to verify equal loading of analyzed samples. Known amounts of purified Cdc34 and ^tm^Cdc34 were used to verify that the α-Cdc34 antibodies have similar affinity for each protein construct.

We next generated a yeast strain in which the wild-type ORF region of *CDC34 *is replaced by an ORF encoding ^tm^Cdc34, leaving the 5' and 3' regions intact [22]. The integration of ^tm^*CDC34 *at the natural chromosomal locus of *CDC34 *supports yeast growth on plates (Figure 2A) and in liquid culture (Figure 2B). In liquid growth medium *CDC34 *(BL2) yeast have an estimated doubling time of 86 +/-2 min and *^tm^CDC34 *(RC85) yeast double every 92 +/- 4 minutes. Apart from this modest difference, these strains have similar cell size and budding profiles, and accumulate to similar cell densities in stationary phase (Figure 2C). Analysis of DNA isolated from dividing *CDC34 *(BL2) and ^t*m*^*CDC34 *(RC85) yeast verifies that they have similar cell cycle profiles under typical laboratory conditions (Figure 2D).

These findings verify that ^tm^Cdc34 protein supports yeast growth despite lacking the catalytic core elements conserved among the Cdc34-like E2s. However, the steady-state level of ^tm^Cdc34 protein in yeast extracts is approximately 4-fold higher than the level of Cdc34 (Figure 2E) even when extracts are prepared by boiling cells directly in SDS-PAGE loading buffer (data not shown). Since the increased steady-state level of ^tm^Cdc34 is seen in a strain isogenic to the *CDC34 *strain, the difference is likely due to a difference in post-translational regulation associated specifically with the replacement of the catalytic E2 motif.

### Purified ^tm^Cdc34 forms an ubiquitin-thiolester and monoubiquitinates substrates, but is defective in substrate polyubiquitination

To address how ^tm^Cdc34 functions without the catalytic core elements conserved among the Cdc34-like E2s, we compared the various activities of purified ^tm^Cdc34 to the activities of wild type Cdc34 and loop-less ^Δ12^Cdc34. The comparison of Cdc34 and ^tm^Cdc34 was done with full-length proteins, but the comparison of all three catalytic cores (wt, *tm*, Δ12) could be performed only with Cdc34 proteins terminated at residue 244 (Figure 1D) because full-length ^Δ12^Cdc34 is insoluble (this work). The previous characterization of ^Δ12^Cdc34 was also done with a C-terminally truncated mutant (^Δ12^Cdc34^270^) [18]. Ectopic expression of Cdc34^244 ^rescues growth of *cdc34Δ *yeast [15,16,27] and supports yeast viability when expressed from the chromosomal location of *CDC34 *[28], demonstrating that the C-terminally truncated form of Cdc34 is functional, at least in the context of the wild type E2 core. All Cdc34 proteins used in this study elute as monomers in gel filtration chromatography (data not shown).

**Figure 3:**
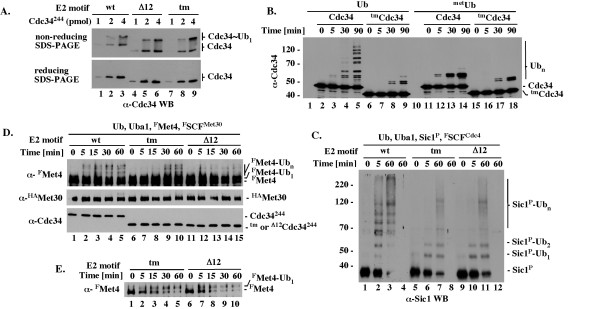
**Purified ^tm^Cdc34 forms ubiquitin thiolester and monoubiquitinates substrates, but is defective in substrate polyubiquitination**. All assays were performed at 25°C in 10-20 μL and included 1 pmol of Uba1 E1 and 1.3 nmol of ubiquitin or its derivative. (A). Formation of ubiquitin-thiolester. The indicated Cdc34^244 ^proteins with wt, tm or Δ12 E2 core (1-4 pmol) were incubated for 5 minutes with Uba1 and ubiquitin followed by SDS-PAGE under reducing (+ βME) or non-reducing (- βME) conditions and α-Cdc34 WB. (B). Autoubiquitination of full length Cdc34 and ^tm^Cdc34. Full length Cdc34 or ^tm^Cdc34 (5 pmol) was incubated for the times indicated with Uba1 and ubiquitin or methylated ubiquitin followed by α-Cdc34 WB. (C). ^F^SCF^Cdc4^-dependent polyubiquitination of Sic1. The indicated Cdc34^244 ^proteins (5 pmol) were incubated for the times indicated with Uba1, the Sic1/Clb5/^Gst^Cdc28 substrate complex (2 pmol) and ^F^SCF^Cdc4 ^(2 pmol) followed by 10% SDS-PAGE and α-Sic1 WB. Reactions shown in lanes 4, 8 and 12 do not have Sic1. (D). ^Gst^SCF^Met30^-dependent polyubiquitination of Met4. Tests were performed as described in C except that with the ^Gst^SCF^Met30 ^E3 and ^F^Met4 substrate. (E). ^Gst^SCF^Met30^-dependent monoubiquitination of ^F^Met4. Tests as in D, but analyzed with shorter (1 instead of 5 minutes) western blot exposure time, which is necessary to visualize unubiquitinated and monoubiquitinated ^F^Met4 as separate species due to similarities in their molecular weights.

We first tested the ability of the purified Cdc34^244^, ^Δ12^Cdc34^244 ^and ^tm^Cdc34^244 ^proteins to form ubiquitin-thiolester. Cdc34 molecules charged with an ubiquitin thiolester are stable only under conditions preventing ubiquitin discharge. Those conditions are ensured by the absence of SCF, substrate and several C-terminal lysines in Cdc34 that serve as intramolecular substrates (Figure 1D) [28]. The charging was performed for a short time (5 minutes) with 1 pmol of Uba1 E1 and 1-4 pmol of Cdc34^244^, ^Δ12^Cdc34^244 ^or ^tm^Cdc34^244 ^followed by SDS-PAGE under either non-reducing conditions (to preserve the thiolester bond) or reducing conditions (to verify that the ubiquitin-thiolester is sensitive to reduction). The charging was not allowed to proceed to completion, thereby ensuring that we could detect an increase or decrease in the percentage of ubiquitin-charged E2 molecules. Under these assay conditions, similar fractions (10-50%) of total Cdc34^244^, ^Δ12^Cdc34^244 ^and ^tm^Cdc34^244 ^molecules are converted into ubiquitin-thiolesters within a range of protein concentrations (Figure 3A). This result agrees with a previous finding that ^Δ12^Cdc34^270 ^forms an ubiquitin-thiolester similarly to wild type Cdc34 [18]. Neither the loop deletion nor the triple mutant alteration of the E2 core thus compromises formation of the ubiquitin-thiolester.

We next tested the ability of full-length Cdc34 and ^tm^Cdc34 to autoubiquitinate one of their C-terminal lysines (Figure 1D). This reaction is inefficient and it is catalyzed only in the absence of SCF [28]. However, due to the intra-molecular nature [28], autoubiquitination provides unique insight into the intrinsic function of Cdc34. We find that autoubiquitination of full-length ^tm^Cdc34 is compromised (Figure 3B, α-Cdc34, compare lanes 6-9 and 1-5). The lack of high molecular weight species in reactions with wild type ubiquitin is consistent with a defect in polyubiquitination of monoubiquitinated ^tm^Cdc34, as it resembles the pattern observed with methylated ubiquitin that cannot be incorporated into polyubiquitin (Figure 3B, α-Cdc34, compare lanes 6-9, 11-14, and 15-18). However, longer reaction times are also needed to detect monoubiquitinated ^tm^Cdc34 in amounts similar to Cdc34 (Figure 3B, α-Cdc34, lanes 11-14 and 15-18). Furthermore, the intra-molecular nature of autoubiquitination [28] does not allow this assay to discriminate between catalytic defects or a change in the intra-molecular contacts between the E2 C-terminus and the E2 core. The altered pattern of autoubiquitination nevertheless provides evidence of a profound intrinsic (independent of SCF) difference between the activities of ^tm^Cdc34 and Cdc34.

We next sought to analyze the activity of ^tm^Cdc34 in the presence of SCF E3. We obtained similar results with full-length ^tm^Cdc34 and C-terminally truncated ^tm^Cdc34^244^. However, only in experiments with C-terminally truncated proteins terminated at residue 244 could we compare the activities of ^tm^Cdc34 and Cdc34 to the activity of ^Δ12^Cdc34 due to the poor solubility of full-length ^Δ12^Cdc34. In SCF^Cdc4^-dependent assays with ^tm^Cdc34^244^, high molecular weight species of the Sic1 substrate (indicative of its polyubiquitination; Sic1-Ub_n_) are visualized via western blot with α-Sic1 antibodies only after long incubation times, exceeding by at least 12-fold (> 60 vs. 5 min) the time necessary to detect Sic1 modified by Cdc34^244 ^(Figure 3C, lanes 5-8, 1-4). ^tm^Cdc34^244 ^is as defective as ^Δ12^Cdc34^244 ^in the formation of high molecular weight Sic1 species and both mutant E2s catalyze a similar accumulation of Sic1 monoubiquitinated on one (Sic1-Ub_1_) or two (Sic1-Ub_2_) lysines (Figure 3C, lanes 5-8, 9-12 and data not shown). Monoubiquitination of additional lysines on Sic1 can be observed in the presence of high Cdc34 concentrations (1-3 μM; [8,9,29,30]) that exceed by about 10-fold the estimated concentration of Cdc34 in cells [31]. We also tested polyubiquitination of ^F^Met4, the SCF^Met30^-dependent substrate modified on a single lysine residue K163 *in vivo *[32] and *in vitro *[33]. Western blot with α-Flag antibodies visualizes polyubiquitinated forms of ^F^Met4 after 5 minutes of reaction with Cdc34^244^, but 30 minutes are needed for similar polyubiquitination by ^tm^Cdc34^244^, suggesting a rate at least 6-fold slower (Figure 3D; α-^F^Met4, lanes 1-5 and 6-10, ^F^Met4-Ub_n_). In contrast, western blot with α-Flag antibodies does not detect polyubiquitinated ^F^Met4 even after 60 minutes of incubation with ^Δ12^Cdc34^244 ^(Figure 3D; α-^F^Met4, lanes 11-15, ^F^Met4-Ub_n_), suggesting that ^Δ12^Cdc34^244 ^is less active than ^tm^Cdc34^244^. This difference reflects a difference in the synthesis of polyubiquitin chain on monoubiquitinated ^F^Met4, as monoubiquitinated ^F^Met4 appears with a similar rate in reactions with ^tm^Cdc34^244 ^and ^Δ12^Cdc34^244 ^(Figure 3E; due to similarities of the apparent molecular weights, unubiquitinated and monoubiquitinated forms of ^F^Met4 are detectable as separate species only under short western blot exposure times). This observation agrees with the finding that deletion of the loop does not affect substrate monoubiquitination by Cdc34 [18].

In summary, ^tm^Cdc34 is normally charged with ubiquitin thiolester and monoubiquitinates substrates in a manner similar to wild type Cdc34 and ^Δ12^Cdc34. However, either the loop deletion or the motif replacement compromises substrate polyubiquitination by Cdc34.

### Unlike in reactions with Cdc34, SCF^Cdc4 ^stimulates the synthesis of free polyubiquitin chains by ^tm^Cdc34 only modestly and in a manner dependent on substrate recruitment

We next addressed how the defect in substrate polyubiquitination by ^tm^Cdc34 relates to the synthesis of free polyubiquitin chains *in vitro*. The *in vitro *synthesis of free polyubiquitin chains by wild type Cdc34 is an acknowledged aspect of its function [18,21,34-36].

**Figure 4:**
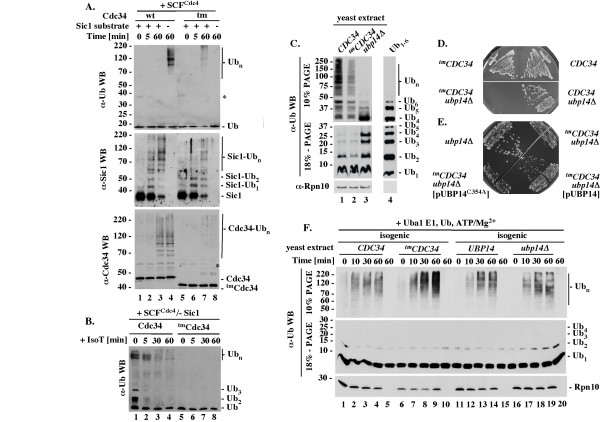
**The synthesis of free polyubiquitin chains *in vitro *and *in vivo***. (A). Regulation of free polyubiquitin chain synthesis by substrate recruitment to SCF^Cdc4^. Standard ubiquitination reactions with Cdc34 or ^tm^Cdc34 (5 pmol) were analyzed by western blots with α-Ub, α-Sic1 or α-Cdc34 antibodies. (B). IsoT sensitivity. Reactions as in A lanes 4 and 8 were analyzed for IsoT sensitivity (Methods). (C). Levels of ubiquitin and ubiquitin conjugates* in vivo*. Boiled cell extracts (Methods) were analyzed by western blot with α-Ub (Covance) or α-Rpn10 antibodies. Lane 4: 200 ng of free polyubiquitin chains Ub_1-6 _purchased from Enzo. (D). Growth of *^tm^CDC34 *but not *CDC34 *yeast is sensitive to loss of *UBP14*. Haploids with the indicated genotypes were selected on haploid selection media with G418 and nourseothricin at 30°C for three days. (E). Overexpression of Ubp14 but not Ubp15^C354A ^supports growth of *^tm^CDC34 upb14Δ *yeast. Heterozygous diploids (RC171 and RC172) were transformed with the indicated plasmids that overexpress Ubp14 (pUBP14) or Ubp14^C354A ^(pUBP14-C354A), patched onto sporulation media and incubated at 26°C for five days. Haploids with the indicated genotypes were selected as in D. (F). Accumulation of ubiquitin conjugates in yeast extracts enriched with Uba1, ubiquitin, ATP and MgCl_2_. Extracts with active ubiquitin-proteasome system (10 μg of total proteins; see Methods) were enriched with Uba1 (10 pmol), ubiquitin (1.3 nmol) ATP (2 mM) and MgCl_2 _(2 mM), incubated at 25°C for the times indicated and analyzed by 10% or 18% SDS-PAGE followed by α-Ub (Sigma) or α-Rpn10 WB.

We first tested the accumulation of free polyubiquitin chains under conditions of a standard ubiquitination assay with wild type Cdc34 and SCF^Cdc4^. Western blot analysis performed with antibodies specific to ubiquitin detects large amounts of high molecular weight polyubiquitin conjugates in the absence but not presence of the Sic1 substrate (Figure 4A, α-Ub WB, lanes 4 and 3). Only the more sensitive western blots with α-Sic1 and α-Cdc34 antibodies can visualize the low amounts of polyubiquitinated Sic1 and Cdc34 present in the same reaction mixtures (Figure 4A, α-Sic and α-Cdc34 WBs). The polyubiquitin conjugates detectable with α-Ub antibodies cannot represent polyubiquitinated Sic1 because they appear in mixtures lacking Sic1 (Figure 4A, compare α-Ub and α-Sic1 WB, lane 4). The polyubiquitin conjugates detectable with α-Ub antibodies cannot represent polyubiquitinated Cdc34, as similar amounts of polyubiquitinated Cdc34 are detectable in the mixtures containing or lacking these conjugates (Figure 4A, compare α-Ub and α-Cdc34 WB, lanes 3 and 4). To verify that these species represent free polyubiquitin chains, we tested whether they are sensitive to Isopeptidase T (IsoT), which disassembles polyubiquitin chains in a manner dependent on the free C-terminus [37]. Most of the conjugates that are detectable by western with α-Ub antibodies disappear upon incubation with IsoT (Figure 4B; α-Ub WB, lanes 1-4), verifying that they represent free polyubiquitin chains. Wild type Cdc34 thus synthesizes super-stoichiometric amounts of free polyubiquitin chains in the presence of SCF^Cdc4 ^and substrate recruitment prevents this process, presumably by re-directing the polyubiquitination activity to substrate.

In contrast, SCF^Cdc4 ^stimulates the synthesis of free polyubiquitin by ^tm^Cdc34 only modestly and in a manner dependent on the Sic1 substrate (Figure 4A, α-Ub WB, compare lane 7 and 8). The ubiquitin conjugates detectable with α-Ub antibodies in reactions with ^tm^Cdc34 likely represent free polyubiquitin chains, because the same α-Ub western blot conditions prevent detection of substrate-attached polyubiquitin chains in reactions even with the more active Cdc34 (described above). We did not verify that the high molecular weight ubiquitin conjugates visualized by α-Ub western blot in ^tm^Cdc34-dependent reactions are sensitive to IsoT, because the necessity for additional dilution in preparation for IsoT treatment (see Methods) prevents their detection.

In summary, Cdc34 synthesizes super-stoichiometric amounts of free polyubiquitin in the presence of SCF^Cdc4 ^and substrate recruitment is sufficient to prevent this process. This regulatory mechanism is disrupted in reactions with ^tm^Cdc34, which synthesizes fewer free polyubiquitin chains and in a manner dependent on, not attenuated by, substrate recruitment.

### *^tm^CDC34 *cells have fewer polyubiquitin conjugates *in vivo*, but their disassembly depends on the Ubp14 C-terminal ubiquitin hydrolase

To test whether ^tm^Cdc34 protein is defective in ubiquitin conjugation *in vivo*, we first performed western blot analysis with α-ubiquitin antibodies of whole cell extracts prepared by direct boiling of *^tm^CDC34 *cells in SDS-PAGE loading buffer. This procedure leads to rapid denaturation of proteins, including proteasome and ubiquitin proteases, and is therefore likely to extract intact ubiquitin conjugates. In these experiments, *^tm^CDC34 *cells have fewer ubiquitin conjugates of a broad molecular weight than *CDC34 *cells (Figure 4C, 10% PAGE, lanes 1 and 2; Ub_4_-Ub_n_). This difference is not accompanied by an accumulation of short polyubiquitin chains and the levels of ubiquitin are similar in both extracts (Figure 4C, 18% PAGE, lanes 1 and 2; Ub_2-4_; Ub_1_). The control western blot performed with α-Rpn10 antibodies verifies that equal amounts of total proteins were analyzed (Figure 4C, α-Rpn10). A reduction in ubiquitin conjugates but not ubiquitin levels is thus detected by analysis of the total ubiquitin conjugates in *^tm^CDC34 *yeast.

The patterns of ubiquitin conjugates detectable in *CDC34 *and, to a lesser degree, in ^t*m*^*CDC34 *cells are similar and involve species of various molecular weights (Figure 4C, 10% PAGE, lanes 1 and 2; Ub_4_-Ub_n_). To assess whether these species represent ubiquitin conjugated to substrates or free polyubiquitin, we sought to test how their pattern changes upon deletion of the *UBP14 *gene, which encodes the only yeast ubiquitin hydrolase that disassembles polyubiquitin in a manner dependent on the free C-terminus [37,38]. In the absence of *UBP14*, free polyubiquitin species would be expected to accumulate in the form of short chains composed of 2-6 ubiquitins, as only longer chains are efficiently disassembled by other DUBs, while the pattern of ubiquitin conjugated to substrates should not change unless the levels of free ubiquitin drop [38]. The high molecular weight ubiquitin conjugates (Ub_n_) detectable in extracts prepared from *CDC34 *yeast are poorly detectable in extracts prepared from *CDC34 upb14Δ *yeast (Figure 4C, 10% SDS-PAGE, Ub_n_, lanes 1 and 3). The loss of ubiquitin conjugates correlates with an appearance of short Ub_2-6 _chains but not a change in ubiquitin level (Figure 4C, 18% SDS-PAGE, Ub_2-6_, Ub_1_, lanes 1 and 3). Free polyubiquitin chains thus represent a prominent fraction of the total ubiquitin conjugates in wild type yeast, at least during vigorous growth. A more accurate estimation is not possible based on these data alone, because western blot detection is not quantitative and the available α-ubiquitin antibodies have different affinities to short and long polyubiquitin chains.

If a significant fraction of the total ubiquitin conjugates detectable in *CDC34 *yeast extracts are free polyubiquitin chains, the lower level of similarly sized ubiquitin conjugates in *^tm^CDC34 *yeast (Figure 4C, 10% SDS-PAGE, lanes 1 and 2) could indicate that *^tm^CDC34 *yeast have fewer free polyubiquitin chains of similar lengths. In this case, fewer short Ub_2-6 _conjugates should be detectable in *^tm^CDC34 *cells lacking *UPB14*. Unexpectedly, this simple prediction could not be tested, because unlike wild type yeast, which are viable in the absence of *UBP14*, the *^tm^CDC34 ubp14Δ *double mutant yeast are inviable (Figure 4D, *^tm^*CDC34 upb14Δ and *CDC34 upb14Δ*). This lethality is corrected by ectopic expression of Ubp14 but not an inactive Ubp14 C354A mutant (Figure 4E), verifying the requirement for Ubp14 function. Thus, *^tm^CDC34 *cells have fewer polyubiquitin conjugates *in vivo*, but these conjugates can be disassembled only by Ubp14.

To get an insight into the Ubp14 dependence, we prepared cell extracts in a manner retaining activity of the ubiquitin-proteasome system (Methods). This procedure is more time consuming than extraction by cell boiling and does not include protease inhibitors. The extracts therefore do not retain the ubiquitin conjugates that were present in cells prior to lysis, leading to similarly low backgrounds in the isogenic *CDC34 *and *^tm^CDC34 *yeast, and in the isogenic *UBP14 *and *ubp14Δ *yeast (data not shown). Instead of inhibiting DUBs, we then attempted to overwhelm their activity by supplementing the extracts with purified Uba1, Ub, ATP and MgCl_2_, which should maximize the ubiquitin conjugation activities of E2s and E3s in the extracts. As expected, the supplementing components alone are insufficient to conjugate ubiquitin (Figure 4F, α-Ub, lanes 5, 10, 15, 20), but stimulate an accumulation of similar amounts of ubiquitin conjugates in the control extracts prepared from *CDC34 *yeast, or the isogenic *UBP14 *or *ubp14Δ *yeast (Figure 4F, 10% PAGE, α-Ub, lanes 1-5, 11-15 and 16-20). We have thus created conditions under which the lack of Ubp14 activity plays no major role in the accumulation of high molecular weight ubiquitin conjugates. Under these conditions, more of high molecular weight ubiquitin conjugates accumulate in ^t*m*^*CDC34 *yeast extract (Figure 4F, α-Ub WB, 10% PAGE, Ub_n_, lanes 1-5, 6-10, 11-15 and 16-20). This effect is repetitive and the control western blot with α-Rpn10 antibodies verifies that equal amounts of total proteins were analyzed (Figure 4F, α-Rpn10).

It cannot be excluded that the 4-fold higher levels of ^tm^Cdc34 protein (Figure 2E) plays a role in the abnormal accumulation of high molecular weight ubiquitin conjugates in *^tm^CDC34 *yeast extract. However, this interpretation would be inconsistent with the defective function of ^tm^Cdc34 *in vivo *(Figure 4C, lanes 1, 2). A more likely explanation is that the polyubiquitin conjugates accumulate due to a delay in their disassembly by DUBs other than Ubp14. If a similar delay characterizes the polyubiquitin conjugates synthesized in *^tm^CDC34 *cells, their disassembly could be primarily dependent on C-terminal hydrolysis, explaining the essential role of Ubp14.

### Phosphorylation of C-terminal serines in ^tm^Cdc34 by Cka2 kinase eliminates the synthesis of free polyubiquitin chains, likely by stimulating their attachment to substrate

**Figure 5:**
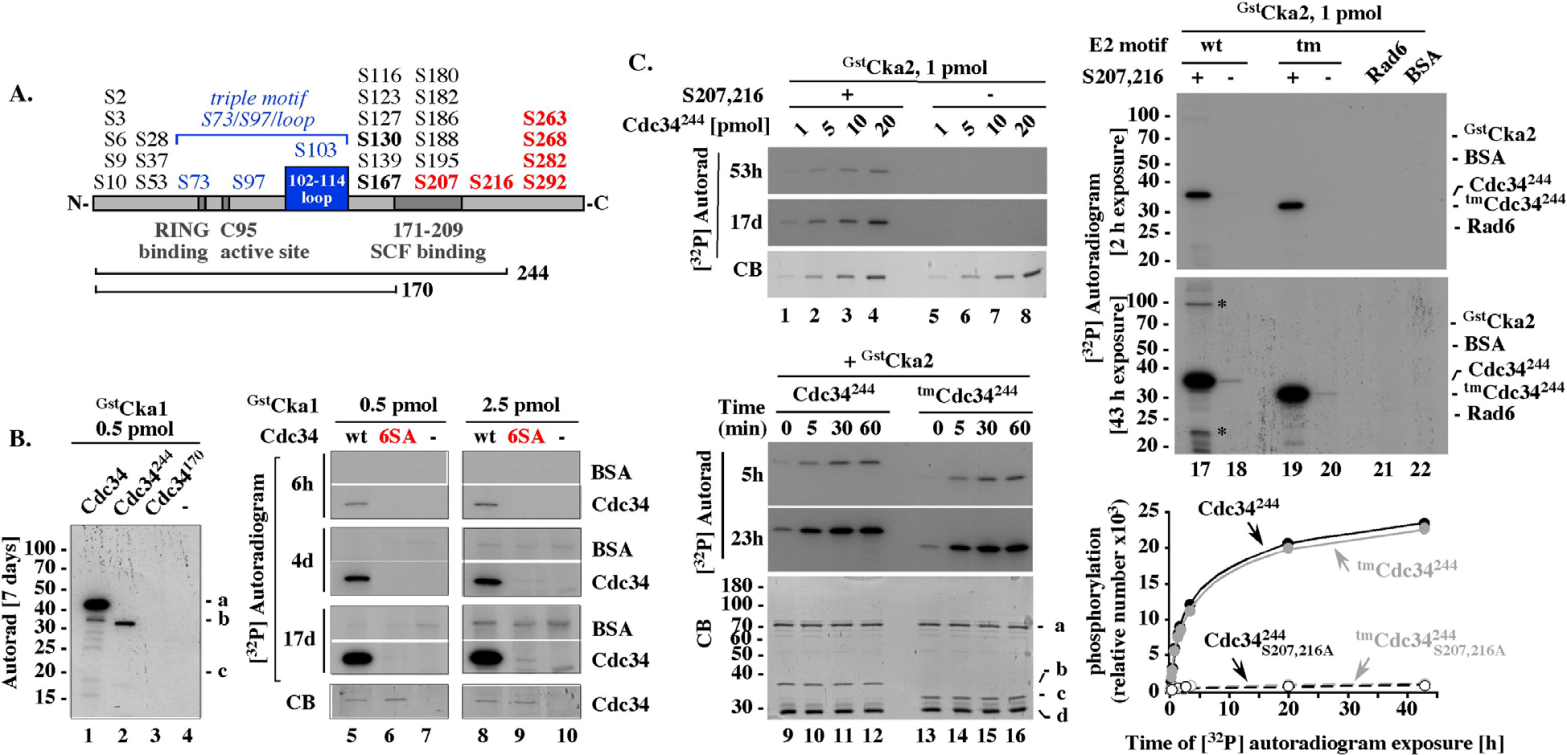
**Yeast ^Gst^Cka1 or ^Gst^Cka2 kinases phosphorylate only the most C-terminal serines in Cdc34 and ^tm^Cdc34**. (A). Scheme of Cdc34. Red: the six most C-terminal serines. Residue 244 represents the C-terminal end in Cdc34^244 ^that includes the E2 catalytic core (a.a. 1 to 170) with the active site cysteine C95, the E3 RING domain-interacting fragment, and the 39 a.a. C-terminal fragment (a.a. 171-209) also implicated in binding to SCF. (B). In a physiological range of protein concentrations, yeast ^Gst^Cka1 phosphorylates only the six most C-terminal serines within Cdc34. 1 hour assays included 10 μM ATP with 0.1 μ Ci of [γ-^32^P]ATP (4500 Ci/mmol), 1 pmol of the indicated Cdc34 constructs and 0.5 or 2.5 pmol of ^Gst^Cka1. (C). ^Gst^Cka2 phosphorylates the S207 and S216 most C-terminal serines in Cdc34^244 ^and ^tm^Cdc34^244^. Assays were performed for 1 hour (or as indicated), with 5 pmols (or as indicated) of the indicated constructs, 1pmol of ^Gst^Cka1 kinase and 10 μM ATP with 5 μCi [^32^P] ATP (4500Ci/mmol), leading to ~50-fold higher sensitivity of [^32^P] detection than in B. Graph: quantitation of the [^32^P] signal of the proteins at different autoradiogram exposure times. CB: Coomassie blue.

Among the genes recently identified as essential for growth of *^tm^CDC34 *but not *CDC34 *yeast is *CKA2 *[22] that encodes the catalytic subunit of casein kinase 2 implicated in Cdc34 function [23,24]. To address how Cka2 affects ^tm^Cdc34 activity, we first tested whether ^Gst^Cka2, or its homologue ^Gst^Cka1, purified from yeast can phosphorylate Cdc34 and ^tm^Cdc34 *in vitro*, and whether the *in vitro *phosphorylation of Cdc34 or ^tm^Cdc34 is limited to the most C-terminal serines [23] (Figure 5A, red), or also targets the catalytic E2 core [24] (Figure 5A, bold black).

Deletion of the C-terminus (Figure 5B, lanes 1-4), or replacement of the six C-terminal serines with alanines (Figure 5B, 6SA; lanes 5-10) prevents phosphorylation of Cdc34 by ^Gst^Cka1. Extended autoradiogram exposure times are required to detect phosphorylation of the Cdc34^6SA ^mutant and this level of phosphorylation is comparable to the control phosphorylation of BSA (Figure 5B, lanes 5-10, 4 and 17 days). Only the six most C-terminal serines are thus phosphorylated within full length Cdc34. Charging Cdc34 with an ubiquitin thiolester under conditions that prevent discharge also does not trigger phosphorylation of the E2 core (data not shown).

Similarly, ^Gst^Cka2 phosphorylates the six most C-terminal serines in Cdc34 (data not shown), or the two most C-terminal serines S207 and S216 in Cdc34^244 ^over a range of concentrations Figure 5C, lanes 1-8). ^Gst^Cka2 phosphorylates Cdc34^244 ^and ^tm^Cdc34^244 ^with similar kinetics (Figure 5C, lanes 9-16), suggesting that the phosphorylation is not affected by the motif replacement. We do not observe phosphorylation of the control Rad6 E2 even under conditions of 50-fold greater sensitivity of [^32^P] detection (Figure 5C, lane 21), verifying that ^Gst^Cka2 function is specific to Cdc34. Only under conditions of such sensitivity, we could detect a low level of phosphorylation of Cdc34^244 ^or ^tm^Cdc34^244 ^carrying the S207A and S216A substitutions (Figure 5C, 43 h, lanes 18, 20). This signal represents ~1% of that associated with phosphorylation of S207 and S216 (Figure 5C, lanes 17, 19 and graph), does not account for quantitative phosphorylation of any other two serines, and was not observed with full-length Cdc34^6SA ^protein (Figure 5B, lanes 5-10).

**Figure 6:**
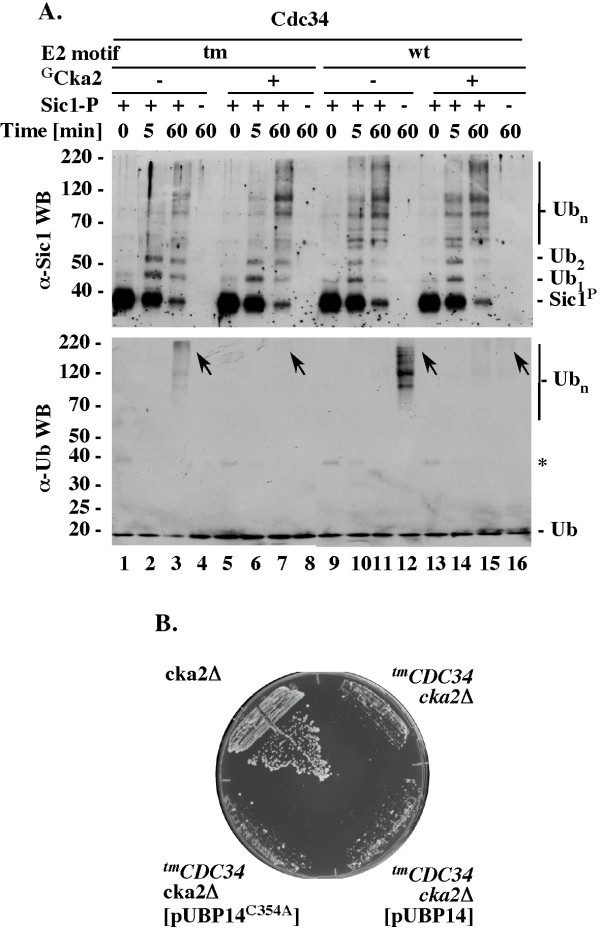
**^Gst^Cka2 inhibits synthesis of free polyubiquitin chains by Cdc34 constructs**. (A). Effects of ^Gst^Cka2 on Sic1 ubiquitination and synthesis of free polyubiquitin chains by Cdc34 proteins. 5 pmol of Cdc34 or ^tm^Cdc34 were phosphorylated at 25°C for 1 hour, as in 5B. The assays were then supplemented with 1 pmol E1, 660 pmol of ubiquitin, 2 pmol of ^Flag^SCF^Cdc4 ^and 2 pmol of Sic1/Cln5/^Gst^Cdc28, where indicated, and incubated at 25°C for the times indicated, followed by α-Sic1 and α-Ub WBs. (B). Ectopic expression of Ubp14 or Ubp15^C354A ^does not rescue growth of *^tm^CDC34 cka2Δ *yeast. The heterozygous diploid RC174 was transformed with the indicated plasmids that overexpress Ubp14 (pUBP14) or catalytically inactive Ubp14 (pUBP14-C354A) from the ADH1 promoter. These transformed diploids as well as RC173 were patched onto sporulation media and incubated at 26°C for five days. Haploids with the indicated genotypes were selected by streaking the heterozygous diploids on haploid selection media with G418 and nourseothricin. Plates were incubated at 30°C for three days.

Phosphorylation of ^tm^Cdc34 by ^Gst^Cka2 kinase modestly stimulates the SCF^Cdc4^-dependent ubiquitination of the Sic1 substrate (Figure 6A α-Sic1 WB, compare lanes 1-4 and 5-8). This effect leads to a nearly normal accumulation of polyubiquitinated Sic1 species in 60 minutes, which is a long reaction time (Figure 6A, α-Sic1 WB, lanes 7, 11 and 15). However, even in the presence of ^Gst^Cka2 fewer polyubiquitinated Sic1 species accumulate in short reactions with ^tm^Cdc34 than with Cdc34 (Figure 6A, α-Sic1 WB, compare lanes 6, 10 and 14). The short reaction times are critical because they represent the time in which substrate degradation needs to be catalyzed *in vivo*. Cka2 thus stimulates, but does not fully rescue, the defective polyubiquitination activity of ^tm^Cdc34. This observation agrees with the finding that *^tm^CDC34 *yeast, which have active Cka2, accumulate fewer polyubiquitin conjugates than *CDC34 *yeast (Figure 4C, lanes 1 and 2).

The stimulation of Sic1 substrate polyubiquitination by ^Gst^Cka2 correlates with a loss of free polyubiquitin chains in the same reaction mixtures (Figure 6A, α-Ub WB, compare lanes 3 and 7, arrows; see Figure 4A and 4B for verification that under the conditions used α-Ub western blot visualizes only free polyubiquitin chains). Phosphorylation of C-terminal serines in ^tm^Cdc34 thus may eliminate the synthesis of free polyubiquitin chains by promoting their attachment to substrate. To assess which of these effects plays a role *in vivo*, we tested whether overexpression of the Ubp14 C-terminal hydrolase rescues viability of *^tm^CDC34 cka2Δ *yeast. Overexpression of Ubp14 or its catalytically inactive mutant does not support growth of *^tm^CDC34 cka2Δ *yeast (Figure 6B), suggesting that the requirement for Cka2 function is not associated with its role in preventing the free polyubiquitin chain synthesis. Rather, it reflects the role in the stimulation of substrate polyubiquitination.

In control reactions with wild type Cdc34, ^Gst^Cka2 has no effect on the SCF^Cdc4^-dependent polyubiquitination of Sic1 substrate (Figure 6A, α−Sic1 WB, compare lanes 9-11 and 13-15). However, ^Gst^Cka2 prevents the synthesis of free polyubiquitin chains in substrate-free reactions with SCF^Cdc4 ^(Figure 6A, α-Ub WB, lanes 12 and 16, green arrows). This effect is detectable only in substrate-free reactions, excluding the possibility that it results from redirecting the synthesis of polyubiquitin chains to substrate. Rather, it reflects a mechanism aimed at inhibiting the unproductive ubiquitin conjugation to free polyubiquitin chains typical of Cdc34 function with substrate-free SCFs. Under conditions of *in vitro *assays performed with low concentration of ubiquitin, or with an access of SCF^Cdc4 ^over substrate, this regulatory effect could indirectly promote substrate polyubiquitination by preventing rapid depletion of free ubiquitin. These possibilities could explain why under some experimental conditions the phosphorylation of wild type Cdc34 could stimulate Sic1 polyubiquitination [23].

## Discussion

Robust polyubiquitination activity is thought to be key to the function of the Cdc34 ubiquitin-conjugating enzyme that, together with the SCF ubiquitin ligases, promotes degradation of proteins involved in cell cycle and growth regulation. However, we find that the ^tm^Cdc34 protein in which the *S73/S97/loop *motif is replaced with the *K73/D97/no loop *motif typical of other E2s supports yeast growth with normal cell size and cell cycle profile despite producing fewer polyubiquitin conjugates *in vitro *and *in vivo*. The significance of this finding comes from the observation that the *in vitro *defect in Sic1 substrate polyubiquitination by ^tm^Cdc34 is similar to the defect of ^Δ12^Cdc34 protein that cannot support growth, and that *^tm^CDC34 *yeast contain fewer polyubiquitin conjugates *in vivo *than *CDC34 *yeast, despite 4-fold higher steady-state levels of ^tm^Cdc34 protein. While several cofactors are implicated in the function of ^tm^Cdc34 *in vivo *[22], these cofactors thus do not ensure normal ubiquitin conjugation in *^tm^CDC34 *yeast.

**Figure 7:**
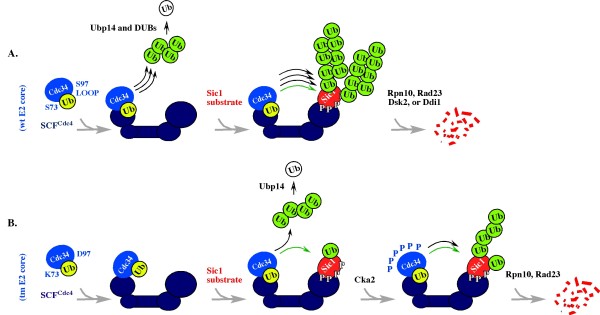
**Summary Model**. The model emphasizes that ^tm^Cdc34 is functional in substrate monoubiquitination but has a defect in the synthesis of substrate-attached and free polyubiquitin chains. This emphasis does not mean that ^tm^Cdc34 cannot synthesize polyubiquitin chains. Rather, it emphasizes that long polyubiquitin chains cannot be synthesized in the short time frame normally available for their synthesis *in vivo*. Cdc34 is marked blue; SCF^Cdc4 ^is marked navy blue; Sic1 substrate is marked red; the free form of ubiquitin (Ub) is marked white; the ubiquitin-thiolester is marked yellow; the ubiquitin engaged in isopeptide bond (with substrate or other ubiquitin molecules) is marked green. Black arrows indicate a robust (three arrows), or modest (one arrow) synthesis of polyubiquitin chains. Green arrow indicates monoubiquitination.

If robust polyubiquitination is not necessary for Cdc34 function *in vivo*, a yet undefined aspect of the SCF-proteasome pathway would be expected to promote substrate proteolysis under conditions of compromised polyubiquitination. In support of this possibility, *^tm^CDC34 *yeast are sensitive to loss of the *RPN10 *and *RAD23 *genes [22] that encode ubiquitin-binding receptors of the proteasome implicated in Sic1 degradation [25,26]. If SCF^Cdc4 ^indeed directly interacts with the proteasome, as one study suggests [39], this interaction could localize ^tm^Cdc34 and substrate to the proximity of the Rpn10 and/or Rad23, thereby compensating for the lack of robust polyubiquitination. This model is outlined in Figure 7. However, it cannot be excluded that the defect in ^tm^Cdc34 is corrected at the proteasome: for example, in a manner dependent on the ubiquitin-interacting motif (UIM) of Rpn10 recently implicated in polyubiquitination [40,41]. An insight into which of these models reflects ^tm^Cdc34 function in *^tm^CDC34 *yeast could come from analysis of the kinetic properties (K_m _and k_cat_) of ^tm^Cdc34 protein *in vitro*, and from testing how Rpn10, and Rad23, individually or as part of the proteasome, affect these properties.

The observation that the catalytic E2 core motifs define distinct mechanisms of function and regulation is consistent with their conservation. However, their analysis in the context of a single E2 that collaborates with only one class of E3s provides the first direct evidence that the E2 motifs control the robustness and regulation of polyubiquitination. What predictions could be made based on this finding for the role and regulation of other E2s? The most obvious prediction is that the alternative motif would support well the function of the E2s that catalyze monoubiquitination, a process that is not related to protein degradation but yet regulates several aspects of cell biology, including endocytosis [42], chromatin remodeling [43] and DNA damage response [44,45]. An example of such an E2 is Rad6, which monoubiquitinates histones and PCNA. The second prediction is that the alternative motif has the potential to support not only monoubiquitination, but also polyubiquitination, at least in reactions with some E3 partners. However, the mechanism of polyubiquitination catalyzed in a manner dependent on each motif would be different. In support of this possibility, two major self-oligomerization schemes linked to an activation of polyubiquitination emerge from analyses of the E2s that differ in their motifs. The Cdc34/Ubc7-like Ube2g2 E2 self-oligomerizes via a RING-independent interaction with its gp78 E3 partner [46]. In contrast, the Ubc5-like E2s, which carry the *K73/D97/no loop *motif, self-oligomerize in a way dependent on the RING domain and non-covalent binding between ubiquitin and several residues located opposite the active site [47-50]. Finally, Cdc34 has a high preference for lysine K48 that is independent of its SCF E3 partner [51-53], but other E2s have lower intrinsic fidelity in lysine selection and frequently collaborate with E3s that define the lysine specificity [53,54]. The low intrinsic fidelity in lysine selection could thus be an evolutionary adaptation that is associated with the *K73/D97/no loop *motif and that allows the synthesis of different linkages depending on the E3 context. In this view, the alternative motif could have evolved to provide a basis for the diversity of polyubiquitin linkages and signaling, while the original motif would have been selected based on the robustness of K48-type of polyubiquitination.

If the motif replacement in Cdc34 lowers the fidelity of lysine selection during polyubiquitin chain synthesis and SCF^Cdc4 ^did not evolve to correct for this change, then polyubiquitin chains synthesized by ^tm^Cdc34 could have altered and/or branched linkages instead of, or in addition to, the K48 type of polyubiquitin typical for Cdc34. This model could explain why ^t*m*^*CDC34 *yeast are sensitive to loss of Ubp14, which is the only DUB that cleaves polyubiquitin in a manner independent of the Ub-Ub linkage [55,56], and why DUBs other than Ubp14 disassemble polyubiquitin conjugates less efficiently when they are produced in *^tm^CDC34 *cell extracts. A similar delay could cause ubiquitin depletion and/or create conditions under which abnormal polyubiquitin chains potently block the proteasome even without massive accumulation [53]. Ubp14 function is not required in yeast [[38], this work], but an inhibition of ubiquitin receptors at the proteasome by an excess of free polyubiquitin [57] is thought to be responsible for the developmental defects associated with Ubp14 absence in multi-cellular organisms, including *D*. *discoideum *[58] and *A. thaliana *[59].

What linkages are present in polyubiquitin chains synthesized by ^tm^Cdc34 protein *in vitro *and *in vivo*, and whether the same linkages are present in substrate-attached and free polyubiquitin are yet unknown. These questions would best be addressed by quantitative mass spectrometry, as ubiquitin mutants could additionally affect the already fragile ^tm^Cdc34/SCF interaction interface (Figure 1B and 1C), which is sensitive to loss of phosphorylation within the C-terminus of ^tm^Cdc34 and does not properly respond to the regulation by substrate recruitment. What is the basis of the functional communication between the seemingly independent E2 and substrate-interacting domains of SCF^Cdc4 ^is yet unknown, but a similar communication is suggested by the observation that substrate recruitment promotes modification of the Cul1 homologue of Cdc53 with the Nedd8 ubiquitin-like protein, leading to E2 activation [60]. Phosphorylation would potentiate the acidic nature of the C-terminus, but how the C-terminus of Cdc34 or ^tm^Cdc34 participates in the mechanism of polyubiquitination is also unclear, as the C-terminus is absent from all known E2 structures (Figure 1C, gray arrow). Why is Cka2, and not Cka1, essential in *^tm^CDC34 *yeast if each kinase phosphorylates the six most C-terminal serines in ^tm^Cdc34 *in vitro*? Cka1 and Cka2 could differ in their times or levels of expression, or localizations, or an undefined feature could give Cka2 a regulatory advantage in *^tm^CDC34 *cells.

Perhaps the most interesting question raised by our study is why the Cdc34/Ubc7-specific motif evolved at all if its replacement in Cdc34 has only a minimal effect on cell growth and division? It is possible that either motif can support Cdc34 function under typical laboratory conditions, but the mechanism defined by the original motif is better for survival under sub-optimal and/or stress conditions. As the only E2 essential for yeast viability and an E2 that has the largest number of substrates known, Cdc34 would likely be required to function under a variety of growth conditions. However, we cannot eliminate the possibility that the motif conservation reflects its ability to undergo post-transcriptional regulation that controls Cdc34 activity and/or levels. This possibility is suggested by the presence of serine residues in the *S73/S97/loop *motif and by the finding that the steady-state level of ^tm^Cdc34 is increased by about four-fold in a strain isogenic to *CDC34 *yeast. This change is significant, as the steady-state levels of Cdc34 do not change during the cell cycle [5] and are unaffected by replacement of all lysines to arginines that prevents autoubiquitination [28]. Furthermore, even a five-fold enrichment of Cdc34 in mammalian cells is sufficient to inhibit an association of CENP-E with kinetochores and to either delay or block metaphase alignment of chromosomes [61,62]; and a similar 4-fold accumulation of Cdc34 has been linked to the development of pediatric T-cell acute lymphoblastic leukemia [63]. If such a modest increase in Cdc34 levels disregulates cell growth and division, a role of the *S73/S97/loop *motif in the control of Cdc34 activity and/or levels could play a role in its conservation. It will be of great interest to determine what aspect of the catalytic E2 motif function and/or regulation is responsible for its conservation.

## Conclusions

Replacement of the catalytic *S73/S97/loop *motif that is conserved among all members of the Cdc34/Ubc7 family with the *K73/D97/no loop *motif present in other E2s compromises the polyubiquitination activity of Cdc34 *in vitro *and *in vivo*, and alters its regulation. However, either motif can support the essential function of Cdc34 in cell growth and division, at least under typical laboratory conditions, raising the question of what is the basis for the motif conservation. We discuss the possibility that growth under suboptimal and/or stress conditions, or a requirement for stringent regulation of Cdc34 levels via the serine residues present in the motif could explain the motif conservation. We predict that future analysis of the cellular pathways that are sensitive to the motif replacement will answer the question of the motif conservation and may define new roles and/or regulatory aspects of Cdc34. The essential role of Cka2, Rpn10 and Rad23 in *^tm^CDC34 *yeast [22] also opens the possibility to define the still enigmatic role of these cofactors in the SCF-proteasome pathway.

## Materials and methods

### Yeast Strains and their construction

All strains and their full genotypes are listed in Table 1. Standard methods were used for strain construction [64]. Strains RC171, RC172, RC173, and RC174 were constructed as described in [22].

**Table 1:** Yeast strains used in this study

Strain	Genotype	Reference
862	*MAT***a ***dsk2::Kan^R ^his3Δ1 leu2Δ0 met15Δ0 ura3Δ0*	[73]

3195	*MAT***a ***ubp14::Kan^R ^his3Δ1 leu2Δ0 met15Δ 0 ura3Δ0*	[73]

13195	*MATα ubp14::Kan^R ^his3Δ1 leu2Δ0 met15Δ0 ura3Δ0*	[73]

BL2	*MAT***a**	[22]

EJ758(YIL035c)	*MAT***a ***his3-Δ200 leu2-3,112 ura3-52 pep4::HIS3 pYEX4T-+rec::CKA1*	[74]

EJ758(YOR061w)	*MAT***a ***his3-Δ200 leu2-3,112 ura3-52 pep4::HIS3 pYEX4T-+rec::CKA2*	[74]

FY24	*MATα ura3-52 trp1-Δ63 leu2-Δ1*	F. Winston

FY78	*MAT***a ***his3Δ200*	F. Winston

KS418	*MAT***a ***CDC34^tm^ura3 leu2 trp1 lys2 ade2 ade3*	[22]

MT1901	*MATα mfa1Δ::pMFA1-HIS3 can1Δ ura3Δ0 leu2Δ0 his3Δ1 lys2Δ0*	M. Tyers

RC6	*MAT***a ***CDC34^tm^(NAT1) ura3 leu2 trp1 ade2 ade3*	[22]

RC21	*MAT***a**/*α CDC34^tm^*(*NAT1*)/*CDC34 ura3/ura3 leu2/leu2 trp1/TRP1 lys2/LYS2 ade2/ADE2 ade3/ADE3 his3/HIS3 MFA1/mfa1Δ::pMFA1-HIS3 can1/CAN1*	[22]

RC29	*MATα cdc34tm(NAT1) mfa1Δ::pMFA1-HIS3 his3Δ ura3Δ leu2Δ can1Δ*	[22]

RC94	*MATαCDC34*(*NAT1*) *mfa1Δ::pMFA1-HIS3 his3Δ1 leu2Δ0 ura3Δ0 can1Δ*	[22]

RC85	*MAT***a ***CDC34^tm^*(*NAT1)*	[22]

RC171	*MAT***a**/*α CDC34^tm^*(*NAT1*)/*CDC34 ubp14::Kan^R^/UBP14 mfa1Δ::pMFA1-HIS3/MFA1 his3Δ/his3Δ ura3/ura3Δ0 leu2/leu2Δ0 can1/CAN1 met15Δ0/MET15*	This study

RC172	*MAT***a**/*α CDC34*(*NAT1*)/*CDC34 ubp14::Kan^R^/UBP14 mfa1Δ::pMFA1-HIS3/MFA1 his3Δ/his3Δ ura3/ura3Δ0 leu2/leu2Δ0 can1/CAN1 met15Δ0/MET15*	This study

RC173	*MAT***a**/*α CDC34^tm^*(*NAT1*)/*CDC34 cka2::Kan^R^/CKA2 mfa1Δ::pMFA1-HIS3/MFA1 his3Δ/his3Δ ura3/ura3Δ0 leu2/leu2Δ0 can1/CAN1 met15Δ0/MET15*	This study

RC174	*MAT***a**/*α CDC34*(*NAT1*)/*CDC34 cka2::Kan^R^/CKA2 mfa1Δ::pMFA1-HIS3/MFA1 his3Δ/his3Δ ura3/ura3Δ0 leu2/leu2Δ0 can1/CAN1 met15Δ0/MET15*	This study

RC175	*MAT***a**/*α CDC34^tm^*(*NAT1*)/*CDC34 rps7b::Kan^R^/RPS7B mfa1Δ::pMFA1-HIS3/MFA1 his3Δ/his3Δ ura3/ura3Δ0 leu2/leu2Δ0 can1/CAN1 met15Δ0/MET15*	[22]

RC176	*MAT***a**/α *CDC34*(*NAT1*)/*CDC34 rps7b::Kan^R^/RPS7B mfa1Δ::pMFA1-HIS3/MFA1 his3Δ/his3Δ ura3/ura3Δ0 leu2/leu2Δ0 can1/CAN1 met15 0/MET15*	[22]

YL10-1	*MAT***a ***cdc34-2 leu2Δ1 ura3-52 trp1Δ63 his3Δ *Gal+	[17]

### Plasmids

see Table 2.

**Table 2:** Plasmids used in this study.

Plasmid	Description	Reference
pYL150	*2μm, LEU2, Amp^r^, GAL10 promoter +CDC34*	[17]

pYL19	*pYL150 with S97D mutation in CDC34*	[17]

pYL27	*pYL150 with 12-residue deletion of G-103 to T-114 in CDC34*	[17]

pYL29	*pYL150 with 12-residue deletion of G-103 to T-114 and S73K and S97D mutations in CDC34*	[17]

pUBP14	*ADH1 promoter and terminator elements + UBP14 in pVT102-U*	[38]

pUBP14-Ala354	*ADH1 promoter and terminator elements +catalytically inactive UBP14(C354A) mutant in pVT102-U*	[38]

### Antibodies

We used α-FLAG M2 and α-Ub (Sigma), α-Ub and α-HA (Covance), α-Cdc34 [28], α-^MBP^Sic1, α-^Gst^Rpn10 and α-^Gst^Skp1 [65]. Antibody detection was by ECL (Amersham).

### Recombinant proteins and protein complexes

We used: apyrase, ubiquitin and methylated ubiquitin (Sigma); Lys48-linked multiubiquitin chains and Isopetidase T (Enzo Research); yeast Uba1^His6 ^[9]; baculoviruses expressing yeast Sic1, Clb5, Cln2^HA^, ^Gst^Cdc28, Cdc53, Rbx1, Cdc4, ^Gst^Skp1, ^Flag^Skp1 [8,10], and ^HA^Met30 [33]; C-terminal His6 fusion (pET21+; Novagen) of full length Cdc34 [29], ^tm^Cdc34 (triple mutant with the *K73/D97/Δ12 *instead of the *S73/S97/loop *motif, this work), Cdc34^6SA ^(with S207, 216, 263, 268, 282 and 292A replacements) [23]; Cdc34^244 ^terminated at residue 244 [29], ^tm^Cdc34^244 ^and ^Δ12^Cdc34^244 ^(this work); ^S207,216A^Cdc34^244^,^ tm,S207,216^Cdc34^244^, ^C95A^Cdc34, ^S97E^Cdc34, Cdc34^170^, Rad6 (this work).

SCF, Sic1 and Cln2 complexes were assembled by co-infecting SF9 insect cells (Invitrogen) for 40 hours with the appropriate baculoviruses, followed by cell lysis and immunoprecipitation [8]. Sic1 was phosphorylated as described previously [8].

All His-tagged proteins were expressed in *E. coli *(BL21 DE3 LysS) and purified on NTA resin (Qiagen) followed by DEAE and HPLC gel filtration chromatography on Superdex 200 (Amersham) in U buffer (50 mM Tris, pH 7.5, 50 mM KCl, 0.2 mM DTT).

^Gst^Cka1 and ^Gst^Cka2 kinases were purified from yeast extracts prepared from cells harvested after 3 hours of treatment with 1 mM CuS0_4 _during logarithmic growth on SD URA medium and blast-frozen in a 1:0.7 ratio of U buffer with 10% glycerol, protease and phosphatase inhibitors. 1 mg of total proteins was diluted to 1 ml with N buffer (50 mM Tris, pH 7.5, 150 mM NaCl, 0.5% Igepal, 0.1 mM DTT with protease and phosphatase inhibitors), incubated at 4°C with 20 μl of G^SH ^Sepharose (Sigma) for 30 minutes, followed by beads wash, equilibration with U buffer and elution with 40 mM gluthation in buffer U, leading to ~10 ng/μl of ^Gst^Cka1 or ^Gst^Cka2.

### Ubiquitination and phosphorylation *in vitro*

Ubiquitination was performed at 25°C for 1 hour or as indicated in 10 μl containing buffer U with 1 mM ATP, 1 mM MgCl_2_, 1 pmol of Uba1, 660 pmols of Ub, 2 pmol of ^Flag^SCF^Cdc4 ^or ^Gst^SCF^Met30 ^(where indicated), 2 pmol of Sic1/Cln5/^Gst^Cdc28 or ^F^Met4 substrate (where indicated), and 1-5 pmol of the indicated Cdc34 proteins as specified in figures. For phosphorylation the assays included buffer U with 10 μM ATP and [^32^P] ATP (ICN) as indicated, the indicated E2 protein constructs, and ^Gst^Cka1 or ^Gst^Cka2. The reactions were analyzed by WB or autoradiography.

### Preparation of yeast extracts with active ubiquitin-proteasome system

Yeast were grown at 30°C in 2 liters of YPD medium [64] starting from OD 0.05 and harvested in the logarithmic phase of growth. Yeast extracts (5-10 mg/ml proteins) were prepared by grinding cells blast-frozen in a 1:0.7 ratio of cells to buffer U (50 mM Tris, pH 7.5; 50 mM KCl; 0.2 mM DTT). The aliquots of extract were frozen in liquid nitrogen and used immediately after thawing. Note that this method does not preserve polyubiquitin conjugates that have been made in cells prior to lysis, because upon cell lysis in the absence of protease inhibitors the polyubiquitin conjugates are either degraded by the proteasome or disassembled by ubiquitin proteases.

### Detection of ubiquitin conjugates in yeast cells

To assess the steady-state levels of ubiquitin conjugates in cells, yeast cells (2.5 × 10^8^) were boiled directly in (150 μl) SDS-PAGE loading buffer with β-mercaptoethanol for 4 minutes at 100°C. Under such conditions, most proteins, including the proteasome and ubiquitin proteases (DUBs) are rapidly inactivated, therefore preserving polyubiquitin conjugates that were present in cells prior to lysis.

### Western blot detection of super-stoichiometric amounts of free polyubiquitin chains

The sensitivity of Western blot detection is restricted by the concentration of the antibodies and the time of their incubation with the antigen-bearing membrane. Western blot performed with high concentrations of α-Ub antibodies (1:100 dilution) and/or for long incubation times (> 2 hr) allows detection of even small amounts of ubiquitin (~100 pmol). Western blot performed with low concentration of α-Ub antibodies (1:5000) and short incubation times (< 30 minutes) can detect only large amounts (μmol) of the same antigen. By varying these parameters, we established western blot conditions that allow detection of polyubiquitin chains only in amounts exceeding the amount of polyubiquitinated Sic1 (2 pmol), Cdc34 (a fraction of 4 pmol) and/or SCF subunits (a fraction of 2 pmol) in the reaction mixtures that were independently verified to contain polyubiquitinated Sic1, Cdc34 and/or SCF subunits via western blots performed with the more sensitive α-Sic1, α-Cdc34, α-Cdc4 or α-Cdc53 antibodies. The inability of western blots with α-Ub antibodies to detect polyubiquitinated Sic1, Cdc34 and/or SCF therefore serves as an internal control, which verifies that the α-Ub Western blots allow detection of only super-stoichiometric amounts of polyubiquitin conjugates. The free nature of these polyubiquitin conjugates is independently verified by their sensitivity to IsoT (see IsoT sensitivity assays).

### Isopeptidase T sensitivity assays

Ubiquitination reactions were performed for 1 hour in the presence of ATP/MgCl_2_, Uba1, Ub, full length Cdc34 and SCF^Cdc4 ^but not Sic1. 50% of the total ubiquitination mixtures were then incubated at 25°C for 10 minutes with apyrase (0.5 unit), which by hydrolyzing ATP prevents function of the Uba1 E1 and stops ubiquitin conjugation, followed by enriched with DTT (10 mM final) and incubation with Isopeptidase T (0.5 mg) for additional 0-30 minutes. The reactions were stopped by boiling with SDS-PAGE loading buffer, separated by SDS-PAGE and analyzed by western blot with α-Ub antibodies.

### Structural modeling

Structural models of the E2 core (a.a. 1-170) of Cdc34 and ^tm^Cdc34 are shown in the context of structures of a scRad6 (gray ribbon representation; PDB ID 1AYZ; [66]) and scUbc7 fragment (gray ribbon representation; PDB ID 2UCZ; [67]). To model the ubiquitin-charged Cdc34 (a.a. 1-170) bound to the RING domain of Rbx1, scUbc7 structure (dark blue; PDB ID 2UCZ; [67]) was first superimposed with the structure of Ubc1 (light blue) charged with ubiquitin (green; PDB ID 1FXT; [68]) and with the structure of hUbc7 (not shown) in complex with the RING of c-Cbl (not shown; PDB ID 1FBV; [69]). The RING domain of c-Cbl was then used to position the RING domain Rbx1 in complex with Cul1 (red and yellow, respectively; PDB ID 1LDK; [70]). Note that the E2 core domain (a.a. 1-170) used for modeling does not include the 39 a.a. (a.a. 171-209) C-terminal fragment unique to Cdc34 that would extend from the alpha helix marked with a gray arrow. The amino acid numbers refer to the position of the residues in Cdc34. Models were done with the ICM program [71].

### Cell cycle distributions

Yeast cells were analyzed by flow cytometry for their DNA content using propidium iodide staining [72].

## Competing interests

The authors declare that they have no competing interests.

## Authors' contributions

AL cloned and purified most of the mutant proteins used in this study, performed most of the *in vitro *tests, and contributed to the conceptual and experimental design of experiments. RC carried the construction and analyses of all yeast strains, and contributed to the conceptual and experimental design of this study. KMS made the initial observation that ^tm^Cdc34 protein has normal monoubiquitination activity, but is defective in polyubiquitination and cloned some ^tm^Cdc34 mutant constructs. MS sub-cloned some Cdc34 and Cdc34^244 ^constructs. SK has performed the structural modeling. MG contributed to the design of this study and edited the manuscript. DS analyzed the IsoT sensitivity assays, performed ubiquitination assays with yeast extracts, was responsible for the conceptual and experimental design of experiments, prepared figures and wrote the manuscrpt. All authors read and approved the final manuscript.
