# Erratum to: The loop-less ^tm^Cdc34 E2 mutant defective polyubiquitination in vitro and in vivo supports yeast growth in a manner dependent on Ubp14 and Cka2

**DOI:** 10.1186/s13008-016-0018-1

**Published:** 2016-10-06

**Authors:** Agnieszka Lass, Ross Cocklin, Kenneth M. Scaglione, Michael Skowyra, Sergey Korolev, Mark Goebl, Dorota Skowyra

**Affiliations:** 1 Edward A. Doisy Department of Biochemistry and Molecular Biology, Saint Louis University School of Medicine, St. Louis, MO 63104 USA; 2 Department of Biochemistry and Molecular Biology, Indiana University School of Medicine, Indianapolis, IN 46202 USA; 3 Dept. of Neurology, University of Michigan Medical School, Ann Arbor, MI USA; 4 Dept. of Microbiology, Washington University School of Medicine, St. Louis, MO USA

## Erratum to: Cell Division 2011, 6:7 DOI 10.1186/1747-1028-6-7

After publication of this study [1], we found that the preparation of several figures did not follow the guidance given in the Instructions for Authors and resulted in unacknowledged modifications. This erratum acknowledges these modifications and shows that they neither affected the interpretation of the data nor the conclusions drawn from the data. The information below is supplementary in nature, as it does not alter or replace any information included in the study [1].

## Supplemental figures

**Supplement to Figure 1E:**
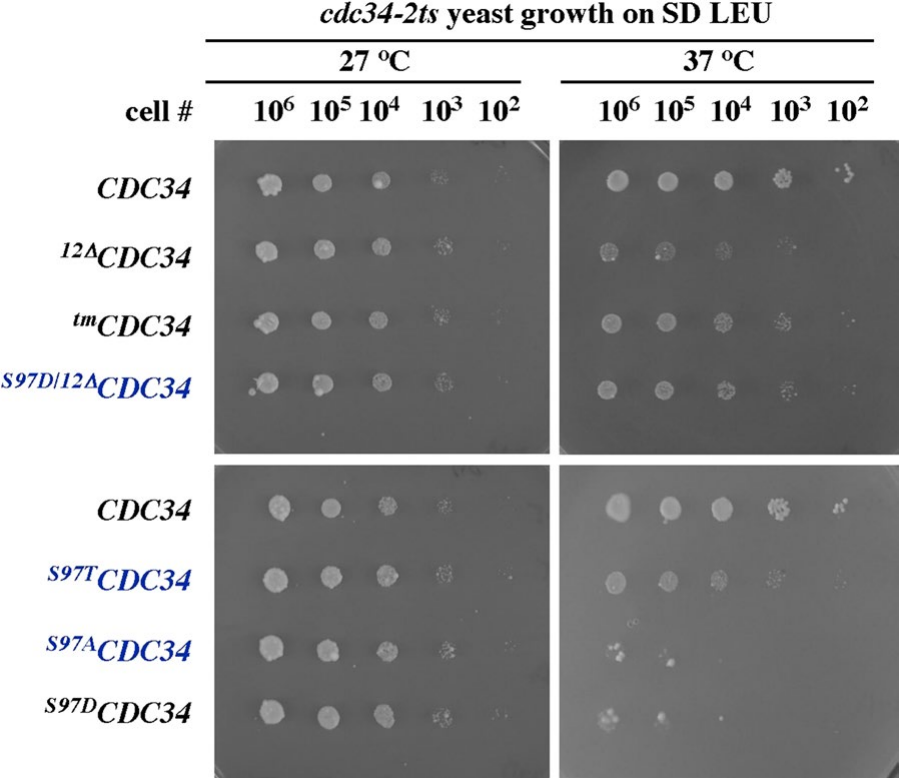
**Original data with** ***cdc34-2ts*** **yeast growth used to prepare Figure 1E [** 1 **]**. Each of the spot-dots shown in Figure 1E [1] was selected from the original electronic image as an individual square and reassembled in the original order with no individual changes in brightness or contrast. This approach allowed us to enlarge the areas with yeast growth without enlarging the empty spaces between spots and, hence, provide more detail. Blue color marks three additional *CDC34* alleles that were not shown in Figure 1E nor discussed in the paper

**Supplement to Figure 2A:**
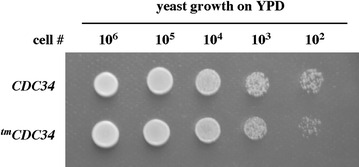
**Original data with yeast growth used to prepare Figure 2A [** 1 **]**. Each spot-dot shown in Figure 2A [1] was selected from the original image as an individual square and reassembled in the original order with no individual changes in brightness or contrast. This approach was consistent with the approach used in Figure 1E, and allowed us to enlarge the areas with growth and, hence, provide more detail

**Supplement to Figure 2C:**
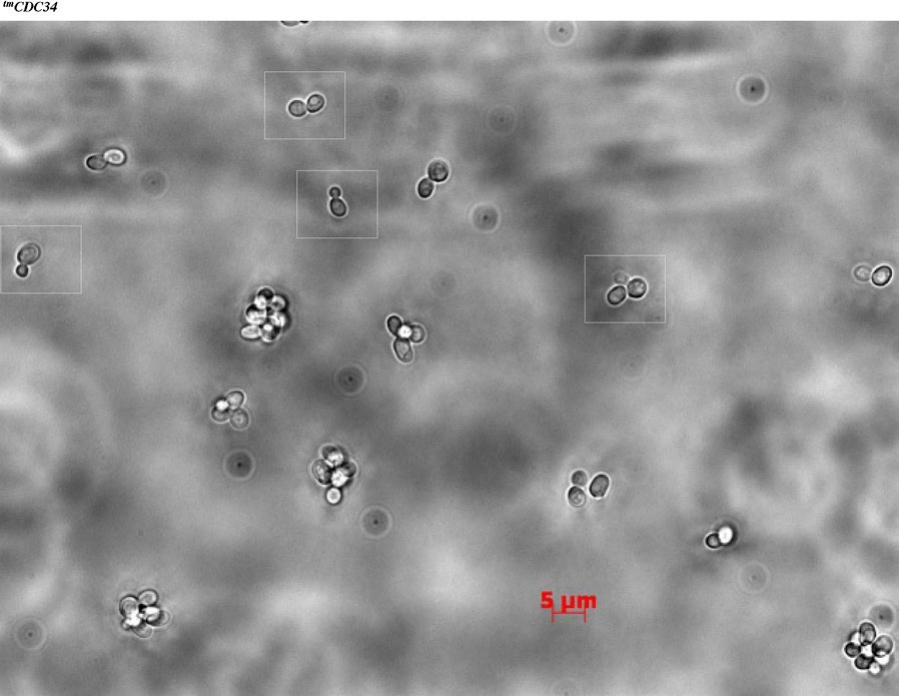
**Original images of** ^**tm**^ ***CDC34*** **cells used to prepare Figure 2C [** 1 **]**. White frames mark the four ^tm^ *CDC34* cells that were intended to be shown. The left two ^tm^ *CDC34* cell images shown in Figure 2C [1] are unintentional duplicates of the same cell

**Supplement to Figure 4A:**
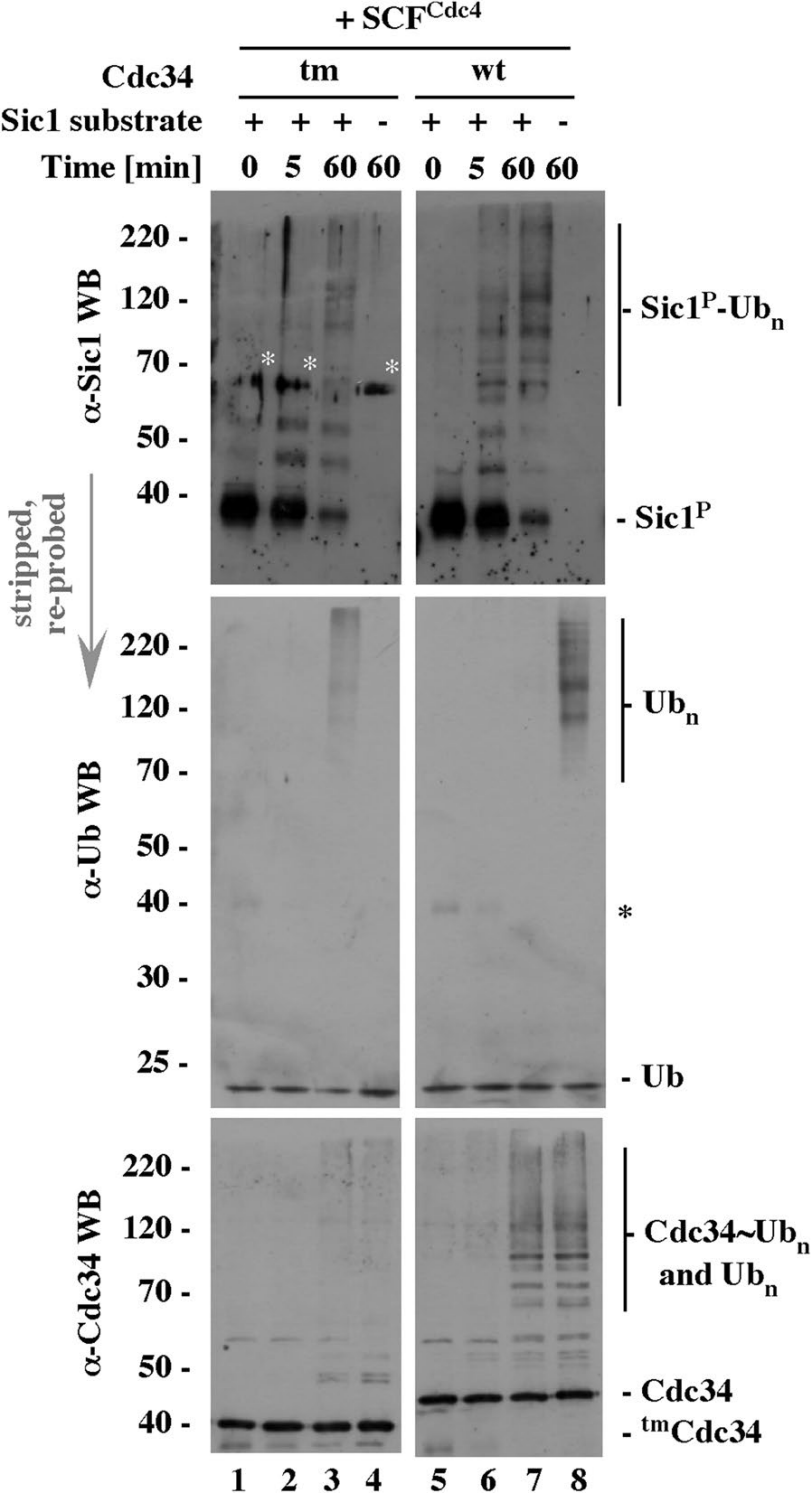
**Original data used to prepare Figure 4A**. ^wt^Cdc34 and ^tm^Cdc34 data sets shown in Figure 4A [1] were selected from a larger set of samples shown in full later in the same report (Figure 6A, [1]). The ^wt^Cdc34 and ^tm^Cdc34 sets of samples shown in Figure 4A [1] were separated on the same gel and analyzed on the same membrane, but were not loaded next to each other, as now indicated by the space between panels. The panels were reassembled without individual changes in brightness and contrast, and are now shown in the original order of loading (^tm^Cdc34 set followed by ^wt^Cdc34 set) and probing (emphasized by gray arrow). The rearranged order shown in Figure 4A [1] was introduced to match the final editorial flow of text, where wtCdc34 samples were described before ^tm^Cdc34 samples (horizontal order), and Ub blots were described before Sic1 and Cdc34 blots (vertical order). This choice of presentation was editorial and had no bearing on the results or their interpretation. White asterisks mark bands of human keratin, a common contaminant cross-reacting with the crude rabbit serum used to detect Sic1, that were inappropriately removed from Figure 4A [1]. The generic label “Sic1 substrate” used on the top of the figure is a synonym of the more specific term Sic1-P (phosphorylated Sic1) used on the right in the α-Sic1 WB panel

**Supplement to Figure 5B:**
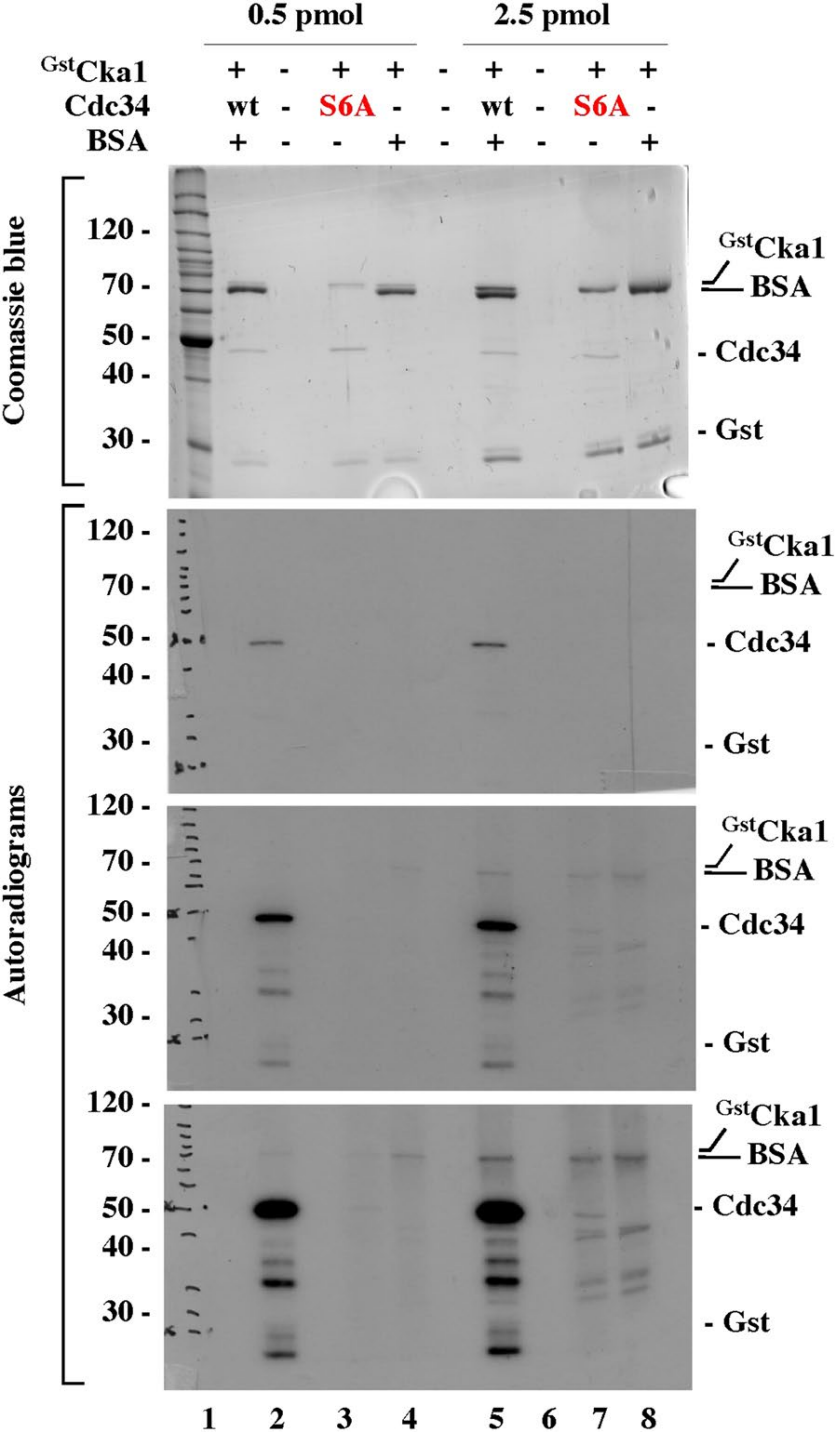
**Original data used to prepare Figure 5B**. Each band shown in Figure 5B [1] was selected from the appropriate part of the original data set as an individual square and reassembled in the appropriate order without individual changes in brightness or contrast. This approach was consistent with the approach used in Figures 1E and 2A, did not alter the identities or the appearances of bands, and was used solely to reduce the overall figure size by removing irrelevant spaces and parts with no factual information

**Supplement to Figure 6A:**
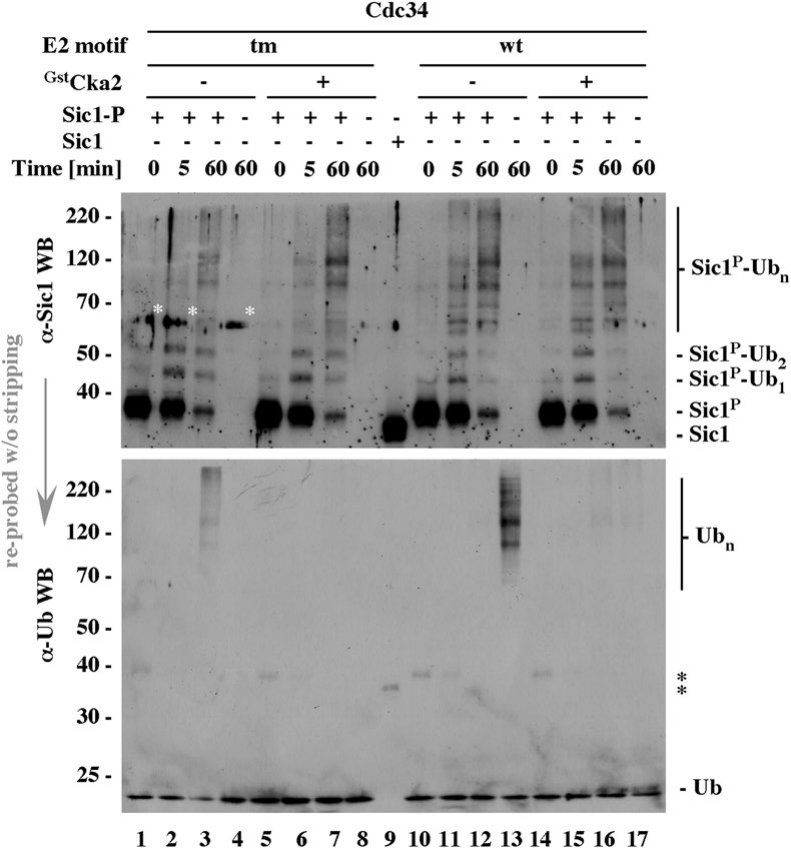
**Original data presented in Figure 6A**. Each of the panels shown in Figure 6A [1] was selected from the same, original, 17-line file and reassembled in the original order without individual changes in brightness or contrast. Panels without Cka2 kinase were first shown separately in Figure 4A [1], to introduce the key differences seen between Cdc34 and ^tm^Cdc34 samples. In addition, the Sic1 image in Figure 6A [1] had two cosmetic alterations. First, three bands of contaminating human keratin, now marked with white asterisks, were inappropriately removed. Second, the control reaction shown in lane 1, one of four identical control reactions shown on the same blot (lanes 1, 5, 10 and 15), was patched to cover up an irrelevant bubble. None of these changes altered the results or their interpretation
